# Modtector: ultra-fast modification signal mining on mapped sequencing reads

**DOI:** 10.1093/bioinformatics/btag627

**Published:** 2026-08-20

**Authors:** Tong Zhou, Yifan Hong, Panfeng Li, Xitong Liu, Jilin Zhang, Xianwei Wang, Ang Li, Lei Sun

**Affiliations:** Shandong Provincial Key Laboratory of Development and Regeneration, School of Life Sciences, Shandong University, Qingdao, 266237, China; Shandong Provincial Key Laboratory of Development and Regeneration, School of Life Sciences, Shandong University, Qingdao, 266237, China; Shandong Provincial Key Laboratory of Development and Regeneration, School of Life Sciences, Shandong University, Qingdao, 266237, China; Shandong Provincial Key Laboratory of Development and Regeneration, School of Life Sciences, Shandong University, Qingdao, 266237, China; Department of Biomedical Sciences, College of Biomedicine, City University of Hong Kong, Hong Kong SAR, 999077, China; Shandong Provincial Key Laboratory of Development and Regeneration, School of Life Sciences, Shandong University, Qingdao, 266237, China; State Key Laboratory of Microbial Technology, Shandong University, Qingdao, 266237, China; Shandong Provincial Key Laboratory of Development and Regeneration, School of Life Sciences, Shandong University, Qingdao, 266237, China; Shandong Provincial Key Laboratory of Development and Regeneration, School of Life Sciences, Shandong University, Qingdao, 266237, China; State Key Laboratory of Microbial Technology, Shandong University, Qingdao, 266237, China

## Abstract

**Summary:**

Existing tools for RNA epitranscriptomic modification and structural signal analysis are often fragmented, inefficiency, and limited to single signal types. We developed Modtector, an unified tool for extracting mutation and reverse-transcription stop signals from aligned sequencing reads. By using a “count-then-correct” strategy, Modtector reduces computational complexity and enables efficient dual-signal analysis. It achieves multi-fold speedups on large-genome and high-coverage datasets, including completing HEK293 22G data analysis in 5 minutes, and show strong scalability on single-cell datasets with speedups exceeding 50-fold.

**Availability:**

The source code is available at GitHub (https://github.com/TongZhou2017/modtector) and Crates.io (https://crates.io/crates/modtector). The archived source-code snapshot used in this study is available at Zenodo (DOI: 10.5281/zenodo.20967747), corresponding to GitHub commit 7c60e9d. Workflow examples, datasets, and analysis scripts are available at Zenodo (DOI: 10.5281/zenodo.17316476 and 10.5281/zenodo.18523297).

## Graphical Abstract

**Graphical Abstract:**
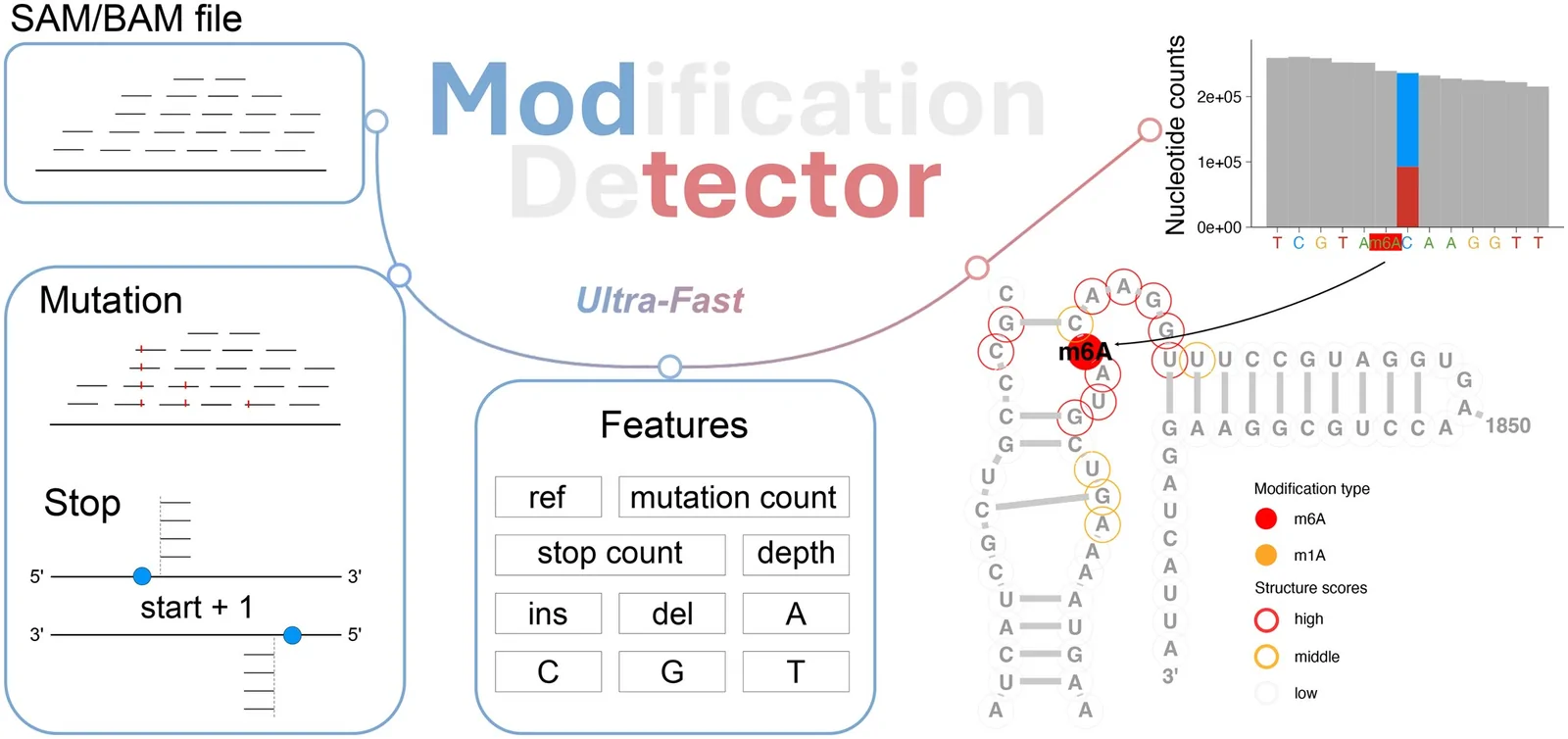

## 1 Introduction

Chemical probing techniques are crucial for studying biomolecular structure and function through high-throughput sequencing (Xu *et al.* 2022, Liu *et al.* 2023). While various computational tools exist for analyzing the resulting signals, most are limited in their ability to jointly process multiple signal types, focusing exclusively on either mutation patterns (Busan and Weeks 2018) or termination events (Li *et al.* 2020), requiring researchers to use separate tools and manually integrate the results, leading to potential inconsistencies and computational overhead.

This fragmentation becomes particularly problematic when analyzing complex modification patterns involving both mutation and stop signals, such as those seen with RNA structure probing (Sexton *et al.* 2017) and m6A modifications (Liu *et al.* 2023). The absence of unified frameworks complicates analytical workflows and hinders direct comparisons between different signal types under consistent statistical conditions (Sexton *et al.* 2017). Furthermore, as sequencing data grows exponentially, computational efficiency has become a key consideration for large-scale analyses (Mu *et al.* 2025). For instance, RASP, which collects over a thousand datasets, struggles to perform genome-wide reanalysis within a reasonable time due to the inefficiency of existing tools (Li *et al.* 2021). Similarly, single-cell data, often generating large volumes of independent datasets for multiple cells, presents challenges for tools like bam-readcount, which is slow and can only process mutation signals, limiting the ability to support future stop signal-based single-cell RNA structural analysis (Wang *et al.* 2024).

To address these challenges, we developed **Modtector (Mod**ification de**tector)**, a unified computational framework that simultaneously extracts and correlates multiple modification signals from aligned sequencing reads. By integrating dual-signal quantification within a single statistical model, Modtector enables researchers to obtain a comprehensive view of modification events in a single execution, ensuring consistent analytical conditions across signal types while delivering superior computational performance.

## 2 Features

### 2.1 Unified framework for dual-signal analysis

**Figure 1:**
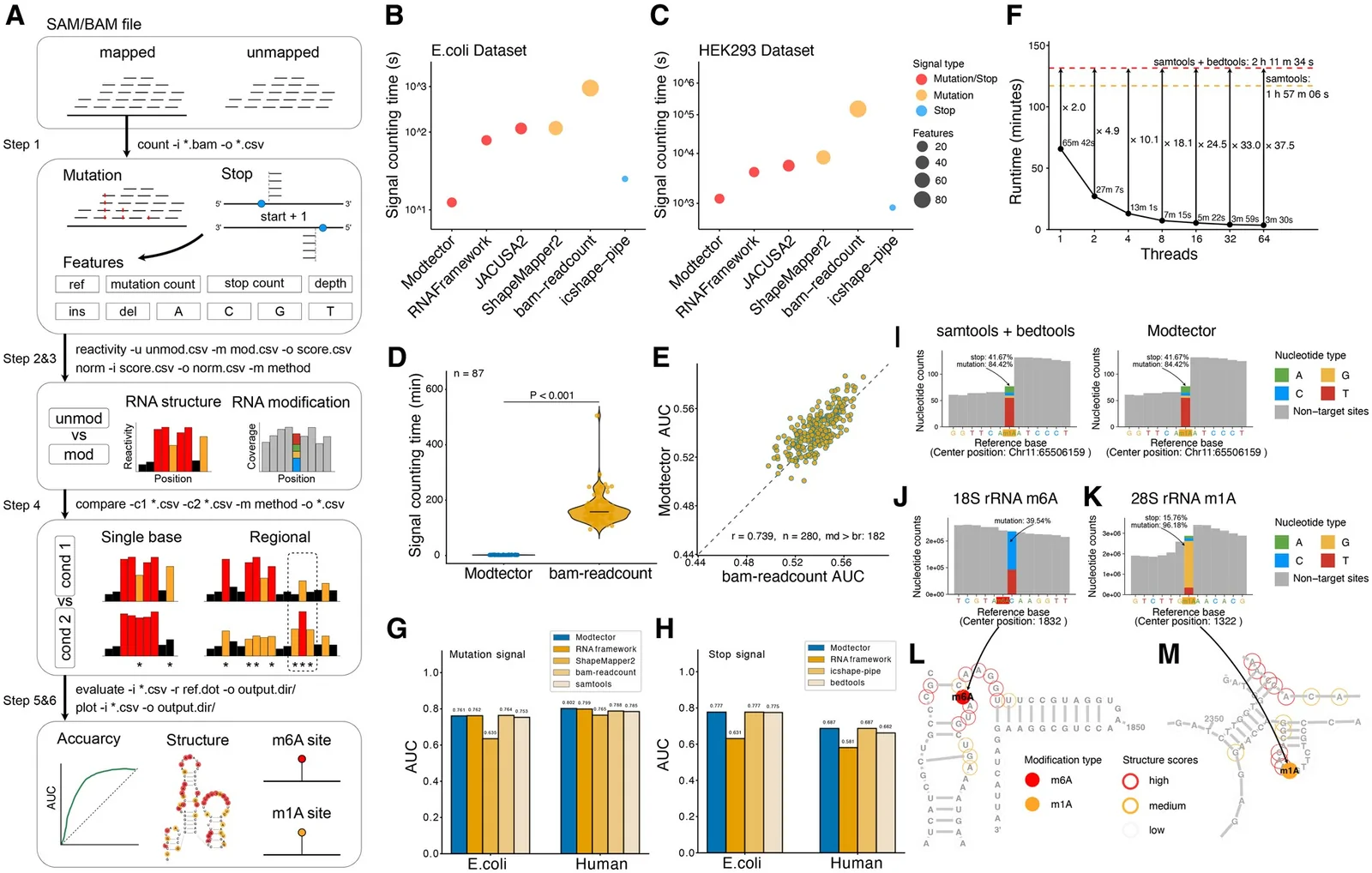
Overview of Modtector’s dual-signal analysis framework and benchmarking results. (A) Workflow of Modtector’s analysis pipeline, illustrating the key steps in processing mapped SAM/BAM files to generate CSV files for mutation and stop signal, applicable to RNA structure and modification data analysis. The flowchart includes command-line examples and key parameters for core functions. (B-C) Runtime comparisons (thread = 1) of Modtector with other tools for RNA structure data analysis using *E. coli* (B) and human HEK293 cell line (C) datasets. Each point represents a tool, with colors indicating the types of signals processed: orange for mutation signals, blue for stop signals, and red for tools supporting both signals. The point size represents the number of features collected. (D) Violin plot comparing runtime efficiency between Modtector and bam-readcount. The mean differences are tested for significance using a paired t-test. (E) Accuracy evaluation for all unmodified (unmod) and modified (mod) paired possibilities of human 18S rRNA. The known RNA structure is sourced from the RNAcentral database (ID: URS0000726FAB_9606). Correlation analysis was performed using Pearson’s method. “Md > br” indicates the number of pairs where Modtector’s AUC exceeds that of bam-readcount. (F) Performance scaling analysis comparing Modtector to samtools and bedtools with varying thread counts (1 to 64). The x-axis represents the number of threads, and the y-axis represents runtime, with log10 scaling applied. Modtector shows significant speed improvements across different thread counts (arrows from data points to the dashed line represent the speedup factor). (G) Comparison of mutation signal–based structure inference accuracy across different RNA structure analysis tools. Results are shown for SHAPE-MaP data (SRR11859184) on *E. coli* 16S rRNA and DMS-MaPseq data (SRR34020745) on human 18S rRNA. (H) Comparison of RT-stop signal–based structure inference accuracy across different RNA structure analysis tools. Results are shown for DMS-seq data (SRR3710405) on *E. coli* 16S rRNA and icSHAPE data (SRR11164863) on human 18S rRNA. (I) Dual-signal distribution of MALAT1 Chr11:65506159 m1A site (m1A-seq dataset: SRR5418425), comparing the consistency between samtools + bedtools and Modtector methods. (J-K) Dual-signal distribution and secondary structure mapping for 18S rRNA 1832 m6A site (J; miCLIP dataset: SRR2003406) and 28S rRNA 1322 m1A site (K; dataset: SRR5418425). The miCLIP method is characterized by mutations or truncations occurring at the previous C position during reverse transcription at m6A sites. (L-M) The secondary structure backbone downloads from the RNAcentral database (L, URS0000726FAB_9606 for 18S rRNA; M, URS000075EC78_9606 for 28S rRNA), and the loop structure scores are mapped through Modtector plot command, with red for high reactivity, yellow for medium reactivity, and grey for low reactivity. Filled circles represent RNA modification sites, with red for m6A modification and orange for m1A modification.

Modtector introduces an integrated pipeline (Fig. 1A) that simultaneously processes two key signal types from SAM/BAM files: nucleotide mismatches (mutation) and reverse transcription truncation events (RT-stop). This unified framework encompasses core functions, including signal counting, reactivity analysis, normalization, evaluation, and visualization, enabling both primary signal calling and downstream analysis methods. Unlike existing tools that typically require separate analyses for each signal type (Table S1), Modtector ensures consistent statistical treatment of both signals by using shared genomic coordinates.

Modtector accelerates signal detection using a “count-then-correct” approach that decouples signal counting and correction, allowing for efficient parallel processing. Traditional methods perform counting and correction sequentially, limiting parallelization, especially for RT-stop signals. Modtector counts mutation and stop signals separately for both forward and reverse strands at each genomic position (Fig. 1A, Step 1), speeding up the process by parallelizing counting across the genome. RT-stop position correction is deferred until after counting, applying a shift based on read alignment, upstream for forward reads and downstream for reverse reads, eliminating the need for read-by-read corrections. By decoupling the computationally expensive counting phase from the correction step, Modtector processes large datasets more efficiently. The counting step runs in parallel across genomic regions without positional dependencies, and runtime improvements scale with larger datasets.

The framework supports both gene-level and genome-level calculations, with genome-wide analysis benefiting from sliding window segmentation and dynamic task deployment to optimize computational resource usage. Overlapping windows (based on stop signal correction lengths) ensure no data loss, addressing issues observed in tools like JACUSA2 (Fig. S1).

For other downstream analysis functions, Modtector offers multiple reactivity calculation and data normalization options. It also provides two distinct approaches for both stop (Ding *et al.* 2014, Rouskin *et al.* 2014) and mutation (Siegfried *et al.* 2014, Zubradt *et al.* 2017) signal (Fig. 1A, Step 2 and 3). Additionally, Modtector supports differential site statistics at both single-base and region [deltaSHAPE (Smola *et al.* 2015) and Diffscan (Yu *et al.* 2022)] levels for different sample groups (Fig. 1A, Step 4). The evaluate function assesses accuracy among base type seperately, providing multiple performance metrics (e.g. AUC, F1-score) to ensure the reliability of the extracted signals (Fig. 1A, Step 5). For the signal score and RNA modification site annotation, Modtector can visualize in secondary structure in SVG format (Fig. 1A, Step 6) and interactive modify (Fig. S2).

### 2.2 Enhanced performance compared to state-of-the-art tools

Comprehensive benchmarking comparing Modtector with leading tools (Table S1) in RNA structure task, such as RNA Framework (Incarnato *et al.* 2018), JACUSA2 (Piechotta *et al.* 2022), ShapeMapper2 (Busan and Weeks 2018), bam-readcount (Khanna *et al.* 2022), and icshape-pipe (Li *et al.* 2020), reveals significant runtime advantages and measured peak memory profiles across representative *E. coli* and human datasets, covering genome-wide and transcript-level analyses as well as RT-stop and mutation signals (Fig. 1B and C and Table S2). Across the six representative resource benchmarks, Modtector achieved the fastest count-step runtime in five datasets and ranked second in the remaining dataset, where icSHAPE-pipe was slightly faster but extracted only four signal dimensions, whereas Modtector simultaneously extracted dual-signal and multi-feature information (Tables S1 and S2). Notably, it demonstrated a speedup of over 100-fold compared to bam-readcount, which is often used for single-cell data analysis (Wang *et al.* 2024), highlighting Modtector’s potential for large-scale single-cell data processing. In a real single-cell dataset test (n = 143), Modtector processed data in an average of 2.96 minutes, achieving a speedup of 50.84-fold over bam-readcount, which took an average of 150.53 minutes (Fig. 1D and E, Fig. S3A and B). This speedup may be partially attributed to the relatively lower sequencing depth of single-cell data, which prevented reaching the full 100-fold improvement.

When compared with classic tools in RNA modification task, Modtector achieved a twofold speedup over conventional samtools (Danecek *et al.* 2021) and bedtools (Quinlan and Hall 2010) pipelines (Fig. 1F). In typical workflows, samtools/bedtools provide predominantly single-threaded core operations and produce intermediate outputs that require additional, user-written scripts to compute extended features, which limits scalability on large-genome, high-coverage datasets. This constraint also indirectly affects tools that depend on these utilities (e.g. RNA Framework; ShapeMapper2). By contrast, Modtector employs a fully Rust-based, unified dual-signal recognition algorithm and leverages multi-threading throughout, including direct bindings to the htslib crate, thereby maximizing computational efficiency and throughput. For example, using only 8 threads, Modtector can process a 22G human dataset in just 5 minutes, whereas other multi-threaded tools require 64 threads to achieve comparable efficiency (Table S2).

Complete benchmarking scripts, commands, software versions, and configuration files are available in Zenodo (DOI: 10.5281/zenodo.18,523,297), with the workflow described in the Supplementary Materials.

### 2.3 Methodological consistency and biological accuracy

In addition to its speed, Modtector demonstrates high methodological consistency and biological accuracy across multiple RNA structure probing datasets (Fig. 1G and H). Modtector achieves consistently high accuracy across both mutation-dominated and RT-stop–dominated datasets, outperforming or matching commonly used tools (Figs. S3C and D and S4). This consistency is further evidenced by Modtector’s ability to produce biologically meaningful RNA secondary and structural mappings (Fig. S4).

In addition, Modtector demonstrates high consistency in RNA modification signal detection. As shown in Fig. 1I, Modtector simultaneously identifies dual-signal distributions at the human lncRNA MALAT1 m1A site (Chr11: 65,506,159). Due to its ability to process both RNA structure and modification signals, Modtector can focus on specific RNA modifications within particular structures, such as the m6A modification on 18S rRNA 1832 (Fig. 1J and L), which is involved in translation regulation (Sepich-Poore *et al.* 2022), or the m1A modification on 28S rRNA 1322 (Fig. 1K and M), which plays a role in ribosomal structure and function regulation (Sharma *et al.* 2018).

To exclude the possibility that the observed dual signals arise from random errors inherent to high-throughput sequencing, we further performed experimental validation (Fig. S5A). At the 28S rRNA m1A1322 site, a pronounced truncated cDNA product was detected (Fig. S5B, lane 2) compared with the AlkB-treated control (Fig. S5B, lane 3). In parallel, Sanger sequencing revealed a mutation-induced mixed G-rich peak at the same position (Fig. S5C and D). These experimental observations are fully consistent with the high-throughput sequencing results (Fig. 1K), confirming that the dual signals identified by Modtector represent genuine RNA modification signatures rather than stochastic sequencing artifacts.

This combination of biological accuracy and methodological consistency makes Modtector a robust and reliable solution for modern chemical probing data analysis.

## 3. Usage and document

Modtector is implemented in Rust and freely available under the MIT License at https://github.com/TongZhou2017/modtector. The software provides a unified command-line interface for simultaneous processing of mutation and stop signals, with subcommands for specialized tasks. Designed for Unix-like environments, the binary executable of Modtector can be directly copied and used without the need for installation, making it easy to integrate into existing bioinformatics workflows. In addition, we tested Modtector in an independent computing environment outside the original development server (Supplementary Materials). Comprehensive documentation, example datasets, and benchmarking scripts are included to facilitate adoption and reproducibility at readthedocs website (https://modtector.readthedocs.io/).

## Supplementary Material

btag627_Supplementary_Data
