# Supplementary material for: Modtector: ultra-fast modification signal mining on mapped sequencing reads
Source: Bioinformatics. 2026 Aug 20;42(9):btag627. doi: 10.1093/bioinformatics/btag627 (PMC13533567; doi:10.1093/bioinformatics/btag627)
Supplement: Supplementary file 1 [file btag627_supplementary_data.zip › Supplementary material.pdf]

Supporting information for

Modtector: Ultra-Fast Modification Signal Mining on Mapped Sequencing Reads

Tong Zhou[1,*,#], Yifan Hong[1,#], Panfeng Li[1,#], Xitong Liu[1], Jilin Zhang[2], Xianwei Wang[1,3], Ang Li[1,*], Lei Sun[1,3,*]

[1] Shandong Provincial Key Laboratory of Development and Regeneration, School of Life Sciences, Shandong University, Qingdao 266237, China

[2] Department of Biomedical Sciences, College of Biomedicine, City University of Hong Kong, Kowloon Tong, Hong Kong SAR 999077, China

[3] State Key Laboratory of Microbial Technology, Shandong University, Qingdao 266237, China

*To whom correspondence should be addressed. #These authors contributed equally to this work.

Contact: tongzhou@sdu.edu.cn (T.Z.); angli41@sdu.edu.cn (A.L.); sunlei0227@sdu.edu.cn (L.S.)

This file includes: Figures S1 to S5, online methods, and independent reproducibility reports.

Methods

Benchmarking workflow and command-line settings

All benchmark analyses were implemented in a Snakemake workflow, and the complete benchmarking scripts, configuration files, and analysis code have been deposited in Zenodo (DOI: 10.5281/zenodo.18523297). The archived Modtector source-code snapshot used in this study is available at Zenodo (DOI: 10.5281/zenodo.20967747), corresponding to GitHub commit 7c60e9d. For all tool comparisons, input reads were processed using the same upstream preprocessing procedure before the counting benchmark. Briefly, raw reads were quality-filtered using fastp v0.23.4, aligned to the corresponding reference using Bowtie2 v2.4.5 in local-alignment mode, and converted to sorted BAM files using samtools v1.17. Where required, BAM files were further filtered by mapping quality (MAPQ ≥ 10). The runtime benchmark focused on the signal-counting step only. Downstream format conversion, reactivity calculation, normalization, visualization, and evaluation were performed after the counting benchmark and were not included in the count-step runtime comparison.

For the counting benchmark, Modtector v0.15.4 was run using *modtector count -b <input.bam> -f <reference.fa> -o <output.csv> -s + --min-base-qual 20*, with the strand parameter adjusted according to the dataset when necessary. bam-readcount v1.0.1 was run using *bam-readcount -f <reference.fa> <input.bam>* with default counting parameters, followed by removal of warning and non-data lines before downstream parsing. RNA Framework v2.9.1 was benchmarked using *rf-count*, RT-stop counting was performed with *rf-count -f <reference.fa> -o <output_dir> -ow <input.bam>*, whereas mutation counting was performed with the *-m* option enabled. ShapeMapper2 v2.2.0 was benchmarked for mutation counting with *-u -m 10 -q 20* on filtered SAM files generated from Bowtie2 alignments. The conventional samtools mutation-counting workflow used mpileup, with minimum base quality and mapping quality both set to 10. The custom RT-stop pipeline used bedtools with 5′-end counting, plus-strand shift −1, minus-strand shift +1. icSHAPE-pipe was benchmarked using *sam2tab* followed by *countRT*, with *sam2tab* run using *-sort yes -ruj yes* and *countRT* using the chromosome-size file generated from

the reference FASTA. Runtime and peak memory usage were recorded using *time -v*, with peak memory reported as the maximum resident set size. The exact commands, file paths, configuration files, and workflow rules used for all benchmarked methods are available in the deposited Zenodo analysis scripts.

Dual-signal experimental validation

To experimentally validate the dual signals identified by Modtector, including both mutation signatures and reverse transcription (RT) stop events, a series of independent verification assays were performed. Putative mutation sites detected computationally were validated by Sanger sequencing of PCR-amplified cDNA fragments encompassing the predicted positions. In parallel, RT stop signals were assessed by gel electrophoresis analysis of reverse transcription products, allowing direct visualization of termination events at nucleotide resolution. Together, these orthogonal experimental approaches provided independent confirmation of the accuracy and reliability of the mutation and RT stop signals inferred by Modtector.

To validate both mutation and RT stop signals, the m1A1322 site of 28S rRNA, a well-characterized modification known to induce RT misincorporation and premature termination, was selected as a representative target. Total RNA and AlkB-treated total RNA were reverse transcribed using SuperScript III Reverse Transcriptase (Thermo Fisher Scientific, USA) with a gene-specific reverse primer (5′-TGGTGCGCCCTCGGCGGACTGGAGA-3′), following the manufacturer's instructions. Following cDNA synthesis, RNA templates were hydrolyzed by the addition of 1 μL of 3 M NaOH and incubation at 90 °C for 12 min, after which the reaction was neutralized with 1 μL of 3 M HCl. The resulting cDNA products were purified and resolved by denaturing gel electrophoresis to assess RT termination patterns and validate RT stop signals at the m1A1322 site.

PCR amplification was performed in a total reaction volume of 50 μL containing 5 μL of cDNA template, 5 μL of 10× reaction buffer, 1 μL of each deoxynucleotide

triphosphate (dNTP; 10 mM; Thermo Fisher Scientific, USA), 1 μL each of the forward primer (5′-CACCCCTCCCACGGCGCGACCG-3′) and RT primer (10 μM), and 0.25 μL of Taq DNA polymerase (New England Biolabs, USA). Amplification was carried out with an initial denaturation at 95 °C for 5 min, followed by 10 cycles of denaturation at 95 °C for 30 s, annealing at 70 °C for 15 s, and extension at 72 °C for 15 s, with a final extension at 72 °C for 5 min. PCR products were resolved by 2% agarose gel electrophoresis, and bands of the expected size were excised, purified, and subjected to Sanger sequencing for mutation validation.

Data Preprocessing

In the data preprocessing stage, quality control of the raw sequencing data is performed using fastp (version 0.20.0) to ensure high-quality input for subsequent analysis. fastp is used to filter low-quality bases, remove duplicate sequences, and eliminate adapter contamination. Sequences with a quality score below a set threshold (typically Q20) are removed. The deduplication option (--dedup) of fastp is applied to remove duplicate sequences introduced during PCR amplification, while the --adapter_fasta option is used to remove any sequencing adapter contamination.

The cleaned data, now free from low-quality reads and contamination, is then prepared for downstream analysis. The sequences are aligned to a reference genome using Bowtie2 (version 2.4.5), with the --local option to enhance the alignment of shorter or incomplete sequences. The alignment results are output in SAM format and subsequently sorted and indexed using samtools, producing BAM files and their corresponding index files. These steps ensure that the data is properly formatted and aligned for use in Modtector.

If needed, mapQ filtering can be applied during the preprocessing stage using samtools, which enables the removal of alignments with low mapping quality. For regions of the genome with exceptionally high sequencing depth, downsampling can be performed using samtools to ensure efficient processing. However, in this study,

no downsampling was performed in order to maximize the comparison of tool performance differences.

Signal Detection and Correction Methods

Modtector utilizes a unified statistical framework to simultaneously detect mutation and reverse transcription (RT) stop signals from sequencing data. This method ensures that both signal types are processed using shared genomic coordinates, allowing for consistent and efficient analysis. The detection and correction process follows a specific sequence: signal counting and signal correction.

Modtector uses the pileup algorithm to efficiently count signals. The pileup approach directly utilizes the original stop signal annotations from the alignment data, rather than iterating over each read individually. This method significantly speeds up the process and forms the basis of Modtector's "count-then-correct" approach.

During signal counting, Modtector distinguishes between reads based on their alignment direction relative to the reference sequence. This read-level strand distinction is important because different correction strategies apply depending on whether a read aligns to the forward or reverse strand of the reference.

Mutation signals are detected by counting nucleotide mismatches between the reference genome and the sequencing reads at each genomic position. The algorithm tracks base counts (A, C, G, T), insertion counts (rf_mutation_ins), deletion counts (rf_mutation_del), and mutation counts (rf_mutation_Count).

Stop signals are identified by detecting reverse transcription truncation events. When a read's start position (head) is encountered in the pileup, it indicates a potential RT stop event. The stop signal is initially recorded at the read's start position, and will be corrected in the next step based on the read's alignment direction.

Once the signals are counted, Modtector applies a "count-then-correct" approach to adjust stop signal positions. This strategy directly avoids the need to process each stop signal at the read level individually. Instead, it leverages pileup's original stop

annotations to quickly correct signals, which is a key factor contributing to Modtector's computational efficiency.

For stop signals, the correction is applied based on the read's alignment direction relative to the reference sequence (determined by the BAM flag indicating whether the read aligns to the forward or reverse strand of the reference). This read-level strand distinction is independent of the genomic strand on which the gene is located. The correction follows theoretical principles:

While reads aligning to forward strand, the stop signal position is corrected by shifting to start_position - 1 (i.e., the position immediately upstream of the read start in the reference coordinate system). This accounts for the fact that RT stops occur at the nucleotide immediately preceding the read start site.

While reads aligning to reverse strand, the stop signal position is corrected by shifting to start_position + 1 (i.e., the position immediately downstream of the read start in the reference coordinate system). This accounts for the reverse transcription directionality when reads are aligned to the reverse complement strand.

The correction formulas are:

For reads aligning to forward strand:

$$P_{corrected} = P_{raw} - 1$$

For reads aligning to reverse strand:

$$P_{corrected} = P_{raw} + 1$$

Where P_{raw} is the read start position detected in the pileup. This read-strand-specific correction adjusts the stop signals to their accurate genomic positions, accounting for the directional nature of reverse transcription and RNA structure. Note that this correction is based on the read's alignment direction, not the genomic strand of the gene, and applies correctly regardless of whether the gene is on the forward or reverse genomic strand.

The output of Modtector is stored in a CSV file format, which contains 13 columns. These columns include the *ChrID*, representing the chromosome or reference sequence identifier, and *pipe_truncation_Strand*, which indicates the strand orientation (with "+" for the forward strand and "-" for the reverse strand). The *pipe_truncation_ChrPos* column provides the corrected genomic position (1-based) of the signal. Additionally, *rf_mutation_Base* records the reference base at the corresponding position, while *rf_mutation_Count* tracks the count of mutation signals, reflecting mismatches between the sequencing reads and the reference. The *pipe_truncation_count* column reports the number of reverse transcription stop signals observed at that position. Other columns include *depth*, which indicates the total read depth at the position, and *rf_mutation_ins* and *rf_mutation_del*, which track the counts of insertion and deletion events, respectively. Finally, the columns *base_A*, *base_C*, *base_G*, and *base_T* report the counts of A, C, G, and T bases observed at the genomic position.

Each row in the output file represents a single genomic position along with its associated signal counts, making it a structured dataset that is ideal for further downstream analysis. This structure is particularly useful for tasks such as visualization, normalization, reactivity calculations, and other interpretations of the detected signals. The CSV format allows for easy integration with additional tools and enables users to conduct comprehensive analyses using the output data.

Reactivity Score Calculation Methods

The reactivity score calculation is a critical step in RNA structure probing analysis, enabling the quantitative assessment of RNA structural accessibility and chemical modification patterns by comparing signal intensities between modified and unmodified samples. The reactivity calculation process transforms raw signal counts into normalized reactivity scores that reflect the structural and chemical properties of RNA molecules at each genomic position.

Modtector introduces an adaptive *k*-factor correction framework that unifies the normalization of both mutation-derived and stop-derived signals across modified and unmodified samples. This framework provides a generalizable strategy for correcting global technical biases while preserving local biological structure signals. In addition to this new adaptive method, Modtector also implements the classical reactivity calculation approaches widely used in RNA structure probing studies, including the Siegfried and Zubradt methods for mutation-based signals and the Ding and Rouskin methods for stop-based signals. To further accommodate emerging experimental designs that omit unmodified controls, Modtector additionally supports a Mod-Only mode with its own mathematical formulation, enabling reactivity estimation directly from a single modified sample.

Reactivity calculation requires CSV-formatted signal count files produced by the Modtector *count* module. A modified-sample CSV file is required, while an unmodified-sample CSV file is optional and used for two-sample correction methods. Each row of the CSV represents a unique genomic position. The first four columns include the chromosome or reference identifier (ChrID), strand orientation (Strand; “+” for forward and “−” for reverse), the 1-based corrected genomic coordinate (Position), and the reference nucleotide (Base).

Signal-related columns include the mutation count (rf_mutation_Count) and the stop count (pipe_truncation_count), which record, respectively, mismatches and reverse transcription termination events at the position. The depth column provides the total number of sequencing reads covering the site. Mutation and stop signal rates are obtained by dividing their respective counts by the depth. Additional auxiliary columns (insertion counts, deletion counts, base-wise nucleotide tallies) may appear in the input file, but these are not directly used in reactivity computation.

Adaptive k-Factor Background Correction method identifies background regions that are expected to exhibit minimal chemical reactivity before computing reactivity scores, typically ribosomal RNA regions (such as 16S or 18S rRNA). These regions provide internal controls from which the program estimates the global ratio of

signal intensities between modified and unmodified samples. For any signal type (mutation or stop), the background regions yield two vectors of signal rates, $\overline{R}_{mod}$ and $\overline{R}_{unmod}$, from which the adaptive correction factor k is calculated as

$$\overline{R} = \frac{1}{n} \cdot \sum_{i=1}^{n} \frac{C_i}{d_i}$$

$$k = \frac{\overline{R}_{mod}}{\overline{R}_{unmod}}$$

This multiplicative factor normalizes overall differences in signal abundance caused by library preparation efficiency, sequencing depth variation, or other technical effects. When no background region is available, or when the mean unmodified signal is zero, the factor defaults to $k = 1$. The adaptive *k*-factor method therefore provides a unified normalization strategy and is applied identically to mutation and stop signals.

For stop signals, Modtector supports two distinct computational methods.

To stabilize variance for high-dynamic-range stop counts, the Ding method applies a logarithmic transform after adding a pseudocount (p). Defining the modified and unmodified raw stop counts as n_{T_i} and n_{U_i}, the transformed quantities are:

$$T_i' = \ln{(n_{T_i} + p)}$$

$$U_i' = \ln{(n_{U_i} + p)}$$

Reactivity is then computed as:

$$R_i = \max{(0, T_i' - U_i')}$$

This method is particularly suitable for datasets with large variance or sparsity. The pseudocount parameter (p) is user-configurable.

If no unmodified sample is provided, stop reactivity is computed directly by Rouskin Method as

$$T_i = \frac{C_i}{d_i}$$

$$R_i = T_i$$

which captures relative accessibility without between-sample normalization.

For mutation signals, Modtector supports two distinct computational methods.

The Siegfried method computes the direct difference of mutation rates:

$$T_i = \frac{C_{mod,i}}{d_{mod,i}}$$

$$U_i = \frac{C_{unmod,i}}{d_{unmod,i}}$$

$$R_i = T_i - U_i$$

This method assumes minimal global biases between samples. Negative reactivity values are allowed, indicating positions where the background mutation rate exceeds the modified sample rate.

In the absence of an unmodified sample, mutation reactivity is defined simply by Zubradt Method as

$$R_i = T_i$$

This formulation is appropriate for modification-only designs or quality-control experiments.

When the user supplies only a modified-sample CSV, Modtector automatically enters Mod-Only mode. In this setting, reactivity is computed exclusively from the modified sample using signal-type-specific single-sample formulas. The reactivity at genomic position (i) for both mutation and stop signals is formally defined as:

$$R_i = T_i$$

No *k*-factor normalization or sample subtraction is applied. The user may further select the Ding method (for stop signals) or similar single-sample computational strategies through the appropriate parameter settings.

Mod-Only mode is activated implicitly when --unmod-csv is omitted. Method selection is controlled via --stop-method and --mutation-method, and pseudocounts for logarithmic calculations are set through --pseudocount.

The output of the reactivity module is a CSV file containing the chromosome identifier, strand, corrected genomic coordinate, and reference nucleotide, followed by two additional columns storing the stop-based and mutation-based reactivity values. The output preserves the row order of the input file to maintain compatibility with downstream steps such as differential reactivity analysis and RNA structure visualization.

Reactivity computations may be customized using parameters that define input file paths, the selected reactivity method for each signal type, the pseudocount used in logarithmic transformations, the number of computational threads, and the path to an optional log file. The modified-sample CSV is specified via --mod-csv, and the unmodified sample via --unmod-csv. Method specification is achieved through --stop-method and --mutation-method, while the Ding pseudocount is configured using --pseudocount.

Normalization methods

Normalization of reactivity profiles is an essential step in RNA structure probing analysis, ensuring that reactivity scores derived from different experimental batches, sequencing depths, or sample preparations are comparable on a unified scale. Modtector provides three normalization algorithms, Percentile28, Winsor90, and Boxplot normalization, that correct for systematic biases by adapting statistical properties of reactivity distributions. An optional piecewise linear transformation based on the Zarringhalam method can further remap the normalized scores to enhance contrast among low-, medium-, and high-reactivity regions.

Normalization operates on reactivity values extracted from CSV files generated by the Modtector reactivity module. Each row corresponds to a single genomic position and comprises the chromosome identifier, strand orientation, genomic coordinate, the stop-derived reactivity value, and the mutation-derived reactivity value. These reactivity columns are processed independently.

Percentile28 normalization, also known as the 2–8% normalization method, rescales reactivity scores using the lower percentiles of the distribution as a reference baseline. For a given reactivity vector $V = \{v_1, \cdots, v_n\}$, the values are first sorted in ascending order. When fewer than ten reactivity values are available within a region, normalization is skipped to avoid instability caused by insufficient sampling. Otherwise, the indices corresponding to the 2nd and 10th percentiles are computed as $p_2 = \lceil 0.02n \rceil$ and $p_{10} = \lceil 0.10n \rceil$, constrained not to exceed$n - 1$. The mean value across the interval $[p_2, p_{10})$ provides the normalization factor:

$$avg = \frac{1}{p_{10} - p_2} \sum_{i=p_2}^{p_{10}-1} v_i$$

where v_i denotes the sorted values. If the interval is empty or the sum is zero, a default factor of 1.0 is applied. Normalized reactivities are obtained by dividing each original value by this mean. This method attenuates the influence of high outliers while retaining global distribution characteristics.

Winsor90 normalization combines Winsorization of the tails with percentile-based scaling. After sorting the vector V, no normalization is applied when only a single value is available. For larger datasets, the 5th and 95th percentile indices are computed as $p_5 = \lfloor 0.05n \rfloor$ and $p_{95} = \lceil 0.95n \rceil$. The threshold values v_5 and v_{95} from the sorted vector act as lower and upper caps, respectively. Winsorization replaces all values below v_5 with v_5, and all values above v_{95} with v_{95}. The Winsorized vector s then normalized by dividing each value by $\max(v_{95}, 10^{-6})$. This approach yields a final reactivity profile bounded between 0 and 1 while remaining robust to extreme outliers.

Boxplot normalization uses the statistical properties of the interquartile range to eliminate extreme outliers before computing a normalization factor. After sorting the

vector, the first (Q1) and third quartiles (Q3) are computed at indices $\lceil 0.25n \rceil$ and $\lceil 0.75n \rceil$, respectively. The interquartile range (IQR = Q3 − Q1) defines an upper bound for outlier exclusion, calculated as $Q3 + 1.5 \times IQR$. Values exceeding this threshold are removed to create a filtered vector $V_{filtered}$, from which the top 10% of values (by ascending order) are extracted and averaged:

$$avg = \frac{1}{k} \sum_{i=m-k}^{m-1} v_{filtered,i}$$

where m is the filtered vector length and $k = \lceil 0.10m \rceil$. This average serves as the normalization factor for all original values. Boxplot normalization emphasizes the upper reactive population while removing extreme aberrant values, making it useful for datasets with strong outliers.

After normalization, an optional piecewise linear transformation may be applied to enhance the interpretability of normalized reactivity scores. For a normalized value x, the Zarringhalam mapping defines four linear segments based on fixed breakpoints:

$$f(x) = \begin{cases} 1.4x, & 0 \leq x < 0.25, \\ 0.35 + 4(x - 0.25), & 0.25 \leq x < 0.3, \\ 0.55 + 0.75(x - 0.3), & 0.3 \leq x < 0.7, \\ 0.85 + 0.5(x - 0.7), & x \geq 0.7. \end{cases}$$

This mapping accentuates differences across reactivity levels while preserving continuity and monotonicity.

Normalization is performed independently for each genomic region, defined by unique combinations of chromosome identifier and strand orientation, to prevent mixing of distinct structural or experimental profiles. Mutation-derived and stop-derived reactivity values are normalized separately because they follow different distributions and may exhibit distinct dynamic ranges. This independence ensures that each signal type is optimally scaled for downstream structural modeling.

The output preserves the input CSV structure, replacing the stop_reactivity and mutation_reactivity columns with normalized values formatted to six decimal places.

Invalid, missing, or undefined values are replaced with zeros. When normalization cannot be performed due to insufficient sample size, the original values are retained.

The normalization method is selected via the --method parameter, supporting Percentile28, Winsor90, and Boxplot normalization. Additional parameters control SNP filtering thresholds, the base types included, window-based normalization behavior, dynamic window sizing, and post-normalization linear mapping. These parameters enable flexible configuration of normalization tailored to the characteristics of specific datasets.

Statistical Algorithms for Differential Site Detection

The statistical algorithms implemented for detecting differential modification sites operate at two distinct levels: single-nucleotide resolution and regional resolution. At the single-nucleotide level, five statistical tests are available: Chi-square test, continuity correction test, Student's t-test, Mann-Whitney U test, and Wilcoxon signed-rank test method. At the regional level, two specialized methods are implemented: DiffScan (with scanning statistics) and DeltaSHAPE (with Z-factor and Z-score analysis). These methods enable robust detection of differentially modified nucleotides and structural regions across different experimental conditions, biological replicates, and sample groups.

RNA structure probing experiments generate quantitative reactivity data that reflect the accessibility of nucleotides and their modification states. Statistical comparison of reactivity values between experimental conditions is essential for identifying differentially modified sites. The statistical algorithms described herein are designed to compare reactivity data at both single-nucleotide and regional resolutions, accounting for experimental variability, biological replicates, and multiple comparison corrections. These algorithms enable the identification of RNA regions with significant changes in reactivity, helping to elucidate structural and functional aspects of RNA molecules.

For single-nucleotide comparisons, the input consists of two sets of reactivity values, corresponding to different experimental conditions or groups. The data for Group 1 and Group 2 are represented as vectors of reactivity scores, denoted $G_1 = \{g_{1_1}, g_{1_2}, \cdots, g_{1_m}\}$ and $G_2 = \{g_{2_1}, g_{2_2}, \cdots, g_{2_n}\}$, where m and n represent the number of replicates or observations in each group, respectively. Each value g_{i_j} corresponds to a reactivity score, RFC (read fraction coverage), or PIPC (proportion of insertions and point mutations) value at a given genomic position.

For regional comparisons, the input data are represented as two series: $S_1 = \{(p_{1_1}, v_{1_1}), (p_{1_2}, v_{1_2}), \cdots, (p_{1_k}, v_{1_k})\}$ and $S_2 = \{(p_{2_1}, v_{2_1}), (p_{2_2}, v_{2_2}), \cdots, (p_{2_k}, v_{2_k})\}$, where p_{i_j} denotes the position, and v_{i_j} represents the reactivity value for that position. The positions in S_1 and S_2 correspond to the same genomic regions, and both series are sorted by position. These series represent contiguous genomic regions, such as specific transcript regions, allowing for comparative analysis of reactivity across regions.

The statistical tests implemented in ModDetector rely on several common parameters to ensure consistency across all methods. One of the key parameters is Alpha (α), which defines the significance level for hypothesis testing and is set to a default value of 0.05. This threshold determines the probability of rejecting the null hypothesis when it is actually true, and it is used to assess the statistical significance of the results. Another important parameter is the Min depth, which serves as a filtering criterion for low-coverage sites. This optional parameter specifies the minimum sequencing depth required for a site to be included in the analysis, ensuring that only well-covered positions are considered, and preventing erroneous results from sites with insufficient data. Additionally, Min fold change is used as another optional filtering threshold to remove sites with negligible changes between experimental groups. This parameter sets the minimum fold change required for a site to be considered as differentially modified, ensuring that only sites with substantial reactivity differences are included in the final analysis. These parameters help control the quality and sensitivity of the statistical tests, allowing users to tailor the analysis to the characteristics of their data and experimental design.

At the single-nucleotide level, various statistical tests are available to compare the reactivity scores between groups. These tests include parametric and non-parametric methods suitable for different experimental designs.

The Student's t-test is a parametric statistical test that compares the means of two independent groups, assuming that the data are normally distributed. This test is suitable when the data are continuous and follow a normal distribution, and when both groups have sufficient sample sizes (typically $n \geq 2$ for each group).

The null hypothesis for the t-test is:

$$H_0 : \mu_1 = \mu_2$$

indicating no difference between the means of the two groups. The alternative hypothesis is:

$$H_1 : \mu_1 \neq \mu_2$$

The steps for computing the t-statistic involve calculating the sample means and variances for both groups, followed by determining the pooled variance. The t-statistic is calculated as:

$$t = \frac{\hat{\mu}_1 - \hat{\mu}_2}{SE}$$

where $\hat{\mu}_1$ and $\hat{\mu}_2$ are the sample means, and SE is the standard error. The degrees of freedom df are computed as $m + n - 2$, where m and n are the sample sizes for Group 1 and Group 2. The p-value is computed using the cumulative distribution function of the t-distribution.

The input includes two vectors of reactivity values, Group 1 and Group 2, each containing at least two observations. The output consists of the t-statistic, p-value, significance result, and effect size (Cohen's d).

The Mann-Whitney U test is a non-parametric method used to compare the distributions of two independent groups. It is suitable for cases where the data are not normally distributed or when sample sizes are small.

The null hypothesis for the Mann-Whitney U test is:

$$H_0 : P(G_1 > G_2) = P(G_2 > G_1)$$

The test computes the U-statistic by ranking the combined data from both groups and calculating the rank sums. The U-statistic is then compared to the expected distribution under the null hypothesis, and a p-value is computed using a normal approximation.

The Wilcoxon signed-rank test is a non-parametric test used for paired samples, comparing the differences between matched observations. It is appropriate for detecting whether the median difference between paired samples is zero.

The null hypothesis for the Wilcoxon signed-rank test is:

$$H_0 : median(G_1 - G_2) = 0$$

The test calculates the differences between paired observations, ranks their absolute values, and computes the rank sums. The test statistic is used to calculate the p-value, and the effect size is determined by the magnitude of the rank differences.

The Chi-square test is a discrete test used to compare observed frequencies to expected frequencies in categorical data. It is typically applied to data that has been discretized into categories, such as reactivity values divided into binary groups.

The Chi-square statistic is calculated as:

$$\chi^2 = \sum_{i,j} \frac{(O_{ij} - E_{ij})^2}{E_{ij}}$$

where O_{ij} is the observed frequency and E_{ij} is the expected frequency. The p-value is calculated from the chi-square distribution, and the significance of the result is determined by comparing the p-value to the chosen alpha level.

At the regional level, specialized methods are implemented for detecting differential modification sites over genomic regions, leveraging both scanning statistics and statistical tests.

The DiffScan method is a region-level statistical approach designed to detect differentially modified RNA regions by identifying clusters of consistent reactivity changes across contiguous genomic positions. Rather than testing individual nucleotides in isolation, DiffScan integrates local, window-based statistical evidence and regional scanning statistics to robustly identify regions exhibiting systematic differences in reactivity between experimental conditions. This strategy is particularly effective for RNA structure probing data, where biologically meaningful signals are often spatially correlated along the transcript.

For a genomic region composed of ordered nucleotide positions $\{p_1, p_2, \cdots, p_k\}$, DiffScan evaluates local reactivity differences by constructing a symmetric sliding window centered at each position p_i. Specifically, for a predefined window radius r, the window is defined as

$$W_i(r) = \{p_i \mid |j - i| \leq r\}$$

with a nominal window size of $2r + 1$, truncated as necessary at region boundaries. Within each window $W_i(r)$, the reactivity values from the two experimental conditions are extracted to form paired vectors representing the local reactivity profiles. These window-level vectors are compared using the Wilcoxon signed-rank test, yielding a position-associated p-value

$$P_i = Wilcoxon(W_i)$$

where P_i reflects the statistical significance of local reactivity differences within the window centered at position p_i. Although indexed by a single nucleotide, each P_i already represents a local regional statistic due to the window-based construction.

To detect extended regions of differential modification, DiffScan aggregates the sequence of window-based p-values using a scanning statistic. For any candidate region $R = [i, i + L - 1]$ of length L, constrained by predefined minimum and maximum region lengths, the regional scanning statistic is defined as

$$Q(R) = \frac{1}{\sqrt{L}} \sum_{j=i}^{i+L-1} -\log(P_j)$$

This formulation emphasizes consecutive small p-values while normalizing for region length, thereby preventing systematic bias toward longer regions. Efficient computation is achieved through prefix sums of $-\log(P_j)$ across the region.

Statistical significance of candidate regions is assessed using a Monte Carlo permutation framework to control for multiple testing across all possible regions. Specifically, the sequence of p-values $\{P_1, P_2, \cdots, P_k\}$ is randomly permuted M times, and for each permutation m, the maximum scanning statistic across all admissible regions is computed as

$$Q_{max}^{(m)} = \max_{R \in \mathcal{R}} Q^{(m)}(R)$$

where $\mathcal{R}$ represents any possible region in the permutation and $Q^{(m)}(R)$ is the scanning statistic for that region. The Monte Carlo simulations are repeated M times to obtain the distribution of the maximum statistic, and the threshold for significance is set at the $(1-\alpha) \times M$ -th permutation, where α is the desired significance level. The threshold is defined as:

$$Q_{threshold} = Quantile_{\alpha}(Q_{max}^{(1)}, Q_{max}^{(2)}, \cdots, Q_{max}^{(M)})$$

where $Quantile_{\alpha}$ represents the $1-\alpha$ quantile of the permutation distribution. The threshold is then used to determine whether a region is significantly modified by comparing the observed scanning statistic $Q(R)$ for each region to the threshold:

$$Q(R) \geq Q_{threshold}$$

Through this hierarchical design, DiffScan explicitly incorporates the window radius rrr at the local testing stage while performing statistical inference at the regional level. This coupling of window-based nonparametric testing with Monte Carlo–controlled scanning statistics enables sensitive and robust detection of differentially modified RNA regions across experimental conditions.

DeltaSHAPE identifies differentially modified regions by calculating the Z-factor and Z-score for each genomic position within a region. This method is designed to detect regions with consistent reactivity differences between experimental conditions.

For each position p_i in the region, the method computes the Delta reactivity ($|\Delta_i| = v_{1_i} - v_{2_i}$) and estimates the error ($\epsilon_{1_i}, \epsilon_{2_i}$) for each sample. The Z-factor and Z-score are calculated as follows:

$$Z_{factor,i} = \begin{cases} 1 - \dfrac{1.96 \times (\epsilon_{1_i} + \epsilon_{2_i})}{|\Delta_i|}, & |\Delta_i| > 0 \\ 0, & |\Delta_i| = 0 \end{cases}$$

$$Z_{score,i} = \frac{|\Delta_i|}{0.1}$$

A position is considered significant if both the Z-factor is greater than 0 and the Z-score exceeds a threshold (typically $|Z_{score}| \geq 1.0$).

Accuracy Evaluation of Reactivity-Based RNA Structure Prediction

The accuracy evaluation framework implemented in ModDetector quantifies the ability of RNA structure probing reactivity data to predict RNA secondary structure by comparing nucleotide-wise reactivity scores against reference structural annotations. The reference structural annotations are sourced from databases such as RNAcentral, with the secondary structure provided in standard formats like dot-bracket notation. The task is formulated as a binary classification problem, where single-stranded nucleotides constitute the positive class and base-paired nucleotides the negative class. Consistent with established probing principles, higher reactivity values are assumed to indicate single-stranded regions.

A key challenge in this evaluation is potential positional misalignment between reactivity measurements and reference secondary structures, arising from annotation offsets, sequence trimming, or experimental artifacts. To address this issue,

ModDetector applies an automatic shift correction procedure that aligns reactivity data with the reference structure by optimizing base-level sequence matching prior to evaluation.

Let the reactivity dataset be denoted as

$$R = \{(p_i, b_i, r_i)\}_{i=1}^{m}$$

where p_i *is the genomic position,* b_i *the observed base, and* r_i *the corresponding reactivity score. The reference secondary structure is represented as*

$$S = \{(p'_j, b'_j, s_j)\}_{j=1}^{n}$$

where $s_j \in \{0,1\}$ *indicates double- or single-stranded state, respectively. An exhaustive search over integer shifts*

$$s \in [-S_{max}, S_{max}], S_{max} = min(|m - n| + 5, 20)$$

is performed, mapping each reactivity position to $p_i + s$. Base matching is evaluated at each shift, treating thymine (T) and uracil (U) as equivalent. The optimal shift is selected by maximizing the total number of matched bases,

$$s^* = arg \max_s \sum_i \mathbb{I}\left(b_i \equiv b'_{p_i+s}\right)$$

where $\mathbb{I}(\cdot)$ *is the indicator function. Using* s^**, matched pairs* $(r_i, s_{p_i+s^*})$ are constructed, while unmatched or out-of-range positions are excluded.

Accuracy evaluation proceeds by thresholding reactivity values to generate binary structural predictions. For a given threshold τ, positions with $r_i \geq \tau$ are predicted as single-stranded, and those with $r_i < \tau$ as double-stranded. This yields a confusion matrix comprising true positives (TP), false positives (FP), true negatives (TN), and false negatives (FN). Standard classification metrics are computed, including sensitivity

$$TPR = \frac{TP}{TP + FN}$$

specificity

$$TNR = \frac{TN}{TN + FP}$$

precision

$$PPV = \frac{TP}{TP + FP}$$

negative predictive value

$$PPV = \frac{TP}{TP + FN}$$

overall accuracy

$$Accuracy = \frac{TP + TN}{TP + TN + FP + FN}$$

and the $\mathrm{F1} - \mathrm{score}$,

$$F1 = \frac{2 \times PPV \times TPR}{PPV + TPR}$$

To characterize performance across decision boundaries, receiver operating characteristic (ROC) and precision–recall (PR) curves are constructed by treating all unique reactivity values as candidate thresholds. For each threshold τ, the false positive rate,

$$FPR(\tau) = \frac{FP(\tau)}{FP(\tau) + TN(\tau)}$$

and the corresponding TPR are computed, yielding ROC points augmented by boundary points (0,0) and (1,1). The area under the ROC curve (AUC) is calculated using the trapezoidal rule,

$$AUC = \sum_{i=1}^{N-1} (FPR_{i+1} - FPR_i) \frac{TPR_i + TPR_{i+1}}{2}$$

PR curves are generated analogously by plotting precision against recall across thresholds.

An optimal classification threshold is selected using Youden's J statistic, which balances sensitivity and specificity while avoiding extreme classification outcomes. Youden's J is defined as:

$$J(\tau) = Sensitivity(\tau) + Specificity(\tau) - 1$$

The optimal threshold is selected by maximizing Youden's J:

$$\tau^* = \arg\max_{\tau} J(\tau)$$

subject to the constraint that thresholds leading to extreme results (where Sensitivity=1.0 and Specificity=0.0, or vice versa) are excluded from consideration. This constraint prevents the selection of degenerate thresholds that classify all samples as a single class. If all candidate thresholds result in extreme outcomes, the method falls back to F1-score maximization:

$$\tau^* = \arg\max_{\tau} F1(\tau)$$

All final metrics are reported at the selected optimal threshold. The Youden's J criterion is particularly effective for binary classification tasks as it directly optimizes the balance between true positive and true negative rates, making it robust to class imbalance while avoiding pathological threshold selections.

In addition to overall evaluation, ModDetector supports base-specific accuracy assessment by stratifying matched positions according to nucleotide identity (A, C, G, and T/U). For each base type with at least ten matched positions, the full evaluation procedure—including ROC analysis and threshold optimization—is performed independently, enabling detection of nucleotide-specific biases. Furthermore, stop-derived and mutation-derived reactivity signals can be evaluated separately using identical procedures, allowing direct comparison of their predictive performance. All numerical metrics and visualizations are reported in standardized formats to facilitate reproducibility and comparative analyses.

Data Visualization Methods for RNA Structure Probing

ModDetector provides a comprehensive suite of visualization tools designed to facilitate the exploration and analysis of RNA structure probing reactivity data. These visualizations, ranging from static charts to dynamic RNA secondary structure mappings, serve to uncover structural insights by representing reactivity patterns and modifications across RNA sequences. The visualization system is equipped to handle

multiple signal types, including stop and mutation signals, enabling in-depth analysis of RNA structural features.

The system facilitates the visualization of raw signal distributions to evaluate data quality across multiple genes. By leveraging line plots and scatter plots, ModDetector can illustrate how stop and mutation signals are distributed across nucleotide positions. These plots are particularly useful for comparing signal intensities between modified and unmodified samples.

In the context of multiple genes, Modtector offers a summary visualization that maps the overall coverage and sequencing depth across all analyzed genes. The X-axis of the scatter plot represents the coverage value (ranging from 0 to the maximum coverage), while the Y-axis shows the logarithmic transformation of average depth, which helps manage the wide range of sequencing depths observed in RNA sequencing data. Points are color-coded based on coverage and depth thresholds, with red indicating high coverage and depth and gray indicating low coverage or depth. This visual approach allows for immediate identification of genes with sufficient coverage for reliable structural analysis, as well as regions that may need further attention due to low sequencing depth.

To visualize reactivity data, which reflects the accessibility of RNA nucleotides, ModDetector generates color-coded bar charts to represent reactivity values at each nucleotide position. The reactivity values are calculated by comparing the stop and mutation signals between modified and unmodified samples. These values are normalized to the range [0, 1], allowing for consistent scaling and easy comparison across regions. The color scheme applied to the bars distinguishes between low, moderate, and high reactivity, aiding in the identification of single-stranded (high reactivity) and double-stranded (low reactivity) regions.

The flexibility of Modtector allows for signal-type-specific visualizations. The system can map stop or mutation signals independently onto RNA structures, making it possible to evaluate the predictive performance of these signals separately. This customization is crucial when comparing the contributions of different signal types to RNA structural understanding, as stop signals and mutation signals may provide

complementary information about RNA structure and modifications. The choice of signal type can be specified through input parameters, ensuring that only relevant data is visualized for the intended analysis. In both cases, the Y-axis represents the normalized reactivity values, and the X-axis corresponds to nucleotide positions across the RNA sequence. These bar charts provide an intuitive means of identifying regions with significant structural changes, which may indicate chemical modifications or accessibility variations in RNA secondary structure.

In addition to overall reactivity evaluations, Modtector provides the option to perform base-specific evaluations. This feature enables separate evaluation of reactivity performance for each nucleotide type (A, C, G, T/U), revealing whether certain bases exhibit different levels of reactivity in the context of RNA secondary structure. This segmentation of reactivity analysis provides insights into the structural properties of each base type and informs the study of base-specific modification patterns.

The evaluation of each base type includes AUC and other evaluation metrics, which are visualized as curve plots to assess how well reactivity data predicts RNA secondary structure for each base. These base-specific evaluations are complemented by overall evaluation metrics generated from the entire dataset, ensuring a holistic view of the reactivity data across all bases.

Beyond static charts, Modtector offers an interactive visualization system that overlays reactivity data on RNA secondary structure diagrams. This feature is designed for in-depth exploration of RNA modifications, enabling the adjustment of visualization parameters such as color thresholds and reactivity ranges in real-time. The interactive interface operates entirely within a local environment, requiring no additional software or external dependencies.

The primary function of this system is to map reactivity scores onto RNA secondary structure diagrams, where reactivity data is represented by color-coded circles placed over the structural diagram. Reactivity data can be mapped for both stop and mutation signals, providing a comprehensive view of RNA structural dynamics. The color

mapping for each nucleotide is based on predefined thresholds that can be adjusted interactively, allowing for customized visualization of reactivity levels.

The interactive system allows zooming and panning across RNA structure diagrams, enabling detailed inspection of specific regions. Zooming adjusts the viewBox based on mouse wheel actions, while panning repositions the structure. The minimap offers an overview of the RNA structure, dynamically updating during zoom or pan, with the ability to quickly navigate by clicking. Additionally, a tooltip system provides real-time nucleotide information, such as position, base type, reactivity, and signal type, when hovering over reactivity circles, enhancing exploration without needing to reference raw data files.

To ensure a responsive and smooth user experience, the interactive visualization system includes several performance optimizations. Event listener management prevents redundant updates, while debounced updates help reduce the frequency of state changes. Conditional rendering ensures that only the visible elements are updated, minimizing computational overhead. The system employs a viewBox-based zoom strategy, which efficiently adjusts the scale and position of the RNA structure without re-rendering the entire structure, resulting in faster interaction with larger datasets.These optimizations ensure that the system can handle both small and large RNA sequences with minimal lag, providing an interactive and fluid exploration experience.

For users who require static images or want to share their visualizations, Modtector includes export functionality that allows for the saving of visualized data in SVG or PNG formats. The SVG export preserves the vector quality of the RNA structure and reactivity overlay, while the PNG export provides a rasterized image, which can be useful for inclusion in publications or presentations. The PNG resolution is adjustable, enabling users to control the quality of the exported image.

Computational Performance Optimization Strategies

The computational performance optimization strategies implemented in Modtector, focusing on dynamic resource allocation, efficiency enhancement methods, and data

format optimization. These strategies ensure efficient processing of large-scale genomic datasets while maintaining data integrity and avoiding redundant computations.

Modtector employs a dynamic resource allocation system that adjusts computational resources based on dataset characteristics, such as size, complexity, and available system resources. This adaptive approach optimizes performance across diverse computational environments and dataset scales.

To estimate the required computational resources, a resource estimation model is used. The required resources R_{req} are calculated as a function of the number of sequencing reads, task complexity, the number of samples, and the memory requirements for processing each sample. The formula for resource estimation is:

$$R_{req} = \alpha \cdot N_{reads} \cdot C_{complexity} + \beta \cdot N_{sample} \cdot M_{memory}$$

where N_{reads} is the number of sequencing reads, $C_{complexity}$ is the task complexity factor, N_{sample} is the number of samples, M_{memory} is the memory required per sample.

The system allocates resources accordingly, determining the optimal number of CPU cores, memory allocation, and batch size for data processing, ensuring that Modtector operates efficiently across datasets of various scales.

Modtector uses a dynamic task deployment system that partitions datasets into optimal chunks based on the available resources and dataset characteristics. This ensures that the computational load is balanced, and memory bottlenecks are avoided.

The chunk size is calculated as:

$$S_{chunk} = \min \left(\frac{M_{available}}{M_{per\_read} \cdot N_{samples}}, \frac{N_{reads}}{N_{cores} \cdot F_{parallel}}\right)$$

where $M_{available}$ is the available memory, M_{per_read} is the memory requirement per read, N_{cores} is the number of CPU cores, $F_{parallel}$ is the parallelization factor.

Once the chunk size is determined, the dataset is divided into smaller, manageable chunks for efficient processing. The optimal number of chunks and their boundaries are calculated based on the available memory and computational resources.

To prevent redundant computations and ensure no data loss, Modtector implements a sliding window approach with overlap management. The window size W_{size} and overlap $W_{overlap}$ are dynamically adjusted based on the dataset and available resources.

The window size is calculated as:

$$W_{size} = \max\left(W_{min}, \frac{N_{reads}}{N_{windows}}\right)$$

where W_{min} is the minimum window size and $N_{windows}$ is the target number of windows. The overlap between windows is calculated as:

$$W_{overlap} = \max\left(O_{min}, \frac{W_{size} \cdot O_{ratio}}{100}\right)$$

where O_{min} is the minimum overlap size, and O_{ratio} is the overlap ratio. The system tracks processed regions to avoid redundant computation, ensuring that only unique regions are processed. This overlap management is crucial for maintaining computational efficiency while ensuring complete data coverage.

In addition to dynamic resource allocation and task deployment, Modtector utilizes several performance optimization strategies to enhance computational efficiency. These include vectorized operations using optimized numerical libraries to reduce computational overhead, and caching of frequently accessed data to prevent redundant computations. Optimized memory management techniques, such as memory pool allocation, are employed to reduce fragmentation and allocation overhead, and the system incorporates garbage collection optimization strategies to maintain efficient memory usage during long-running tasks.

For large datasets, Modtector leverages columnar data formats to improve query performance and processing speed by allowing the system to read only the necessary columns. The system also applies compression techniques to reduce storage space

and I/O overhead, selecting appropriate algorithms based on data type and characteristics. To further reduce memory footprint, Modtector performs streaming data processing so that only small portions of the data are kept in memory at any given time, thereby optimizing memory usage during large-scale genomic analyses.

Figure Legends

Figure 1. Overview of Modtector's dual-signal analysis framework and benchmarking results.

(A) Workflow of Modtector's analysis pipeline, illustrating the key steps in processing mapped SAM/BAM files to generate CSV files for mutation and stop signal, applicable to RNA structure and modification data analysis. The flowchart includes command-line examples and key parameters for core functions.

(B-C) Runtime comparisons (thread = 1) of Modtector with other tools for RNA structure data analysis using *E. coli* (B) and human HEK293 cell line (C) datasets. Each point represents a tool, with colors indicating the types of signals processed: orange for mutation signals, blue for stop signals, and red for tools supporting both signals. The point size represents the number of features collected.

(D) Violin plot comparing runtime efficiency between Modtector and bam-readcount. The mean differences are tested for significance using a paired t-test.

(E) Accuracy evaluation for all unmodified (unmod) and modified (mod) paired possibilities of human 18S rRNA. The known RNA structure is sourced from the RNAcentral database (ID: URS0000726FAB_9606). Correlation analysis was performed using Pearson's method. "Md > br" indicates the number of pairs where Modtector's AUC exceeds that of bam-readcount.

(F) Performance scaling analysis comparing Modtector to samtools and bedtools with varying thread counts (1 to 64). The x-axis represents the number of threads, and the y-axis represents runtime, with log10 scaling applied. Modtector shows significant speed

improvements across different thread counts (arrows from data points to the dashed line represent the speedup factor).

(G) Comparison of mutation signal–based structure inference accuracy across different RNA structure analysis tools. Results are shown for SHAPE-MaP data (SRR11859184) on *E. coli* 16S rRNA and DMS-MaPseq data (SRR34020745) on human 18S rRNA.
(H) Comparison of RT-stop signal–based structure inference accuracy across different RNA structure analysis tools. Results are shown for DMS-seq data (SRR3710405) on *E. coli* 16S rRNA and icSHAPE data (SRR11164863) on human 18S rRNA.
(I) Dual-signal distribution of MALAT1 Chr11:65506159 m1A site (m1A-seq dataset: SRR5418425), comparing the consistency between samtools + bedtools and Modtector methods.
(J-K) Dual-signal distribution and secondary structure mapping for 18S rRNA 1832 m6A site (J; miCLIP dataset: SRR2003406) and 28S rRNA 1322 m1A site (K; dataset: SRR5418425). The miCLIP method is characterized by mutations or truncations occurring at the previous C position during reverse transcription at m6A sites.
(L-M) The secondary structure backbone downloads from the RNAcentral database (L, URS0000726FAB_9606 for 18S rRNA; M, URS000075EC78_9606 for 28S rRNA), and the loop structure scores are mapped through Modtector plot command, with red for high reactivity, yellow for medium reactivity, and grey for low reactivity. Filled circles represent RNA modification sites, with red for m6A modification and orange for m1A modification.
Alt text: Overview of the Modtector workflow and benchmarking results, showing dual-signal extraction from mapped reads, runtime comparisons with existing tools, accuracy evaluations, and examples of RNA modification signal visualization.

Figure S1. Expanded resource benchmark across genome-wide and transcript-level datasets.
(A) JACUSA2 loses data in multi-thread models due to overlapping signal corrections. The x-axis represents the number of threads (1 to 64), and the y-axis represents the number of output file rows.

Alt text: Resource and consistency benchmark for additional analyses, showing how output rows or resource metrics vary across tools, thread settings, and representative genome-wide or transcript-level datasets.

Figure S2. Interactive visualization platform for RNA secondary structure.
(A) Overall interface layout.
(B) Basic style control panel.
(C) Reactivity score threshold adjustment panel.
(D) Single-site style editing function.
(E) SVG data import module.
(F) Export function for result figures.

Alt text: Screenshots of the Modtector interactive RNA secondary structure visualization platform, showing the main interface, style controls, reactivity threshold adjustment, single-site editing, SVG import, and figure export functions.

Figure S3. Runtime and accuracy evaluation on a single-cell dataset.
(A-B) Violin plot comparing runtime efficiency between Modtector and bam-readcount. The mean differences are tested for significance using a paired t-test.
(C-D) Accuracy evaluation for all unmodified (unmod) and modified (mod) paired possibilities of human 18S rRNA. The known RNA structure is sourced from the RNAcentral database (ID: URS0000726FAB_9606). Correlation analysis was performed using Pearson’s method. "Md > br" indicates the number of pairs where Modtector's AUC exceeds that of bam-readcount.

Alt text: Single-cell benchmark comparing Modtector and bam-readcount, showing runtime distributions and accuracy comparisons based on human 18S rRNA structure-derived evaluation.

Figure S4. Visualization of SSU rRNA secondary structure with reactivity scores from different tools.

Mutation-based reactivity scores from SHAPE-MaP (A; SRR11859184) and DMS-MaPseq (B; SRR34020745), as well as RT-stop–based reactivity scores from DMS-seq (C; SRR3710405) and icSHAPE (D; SRR11164863), calculated by Modtector and other tools, are mapped onto RNA secondary structures using the Modtector plotting module. Results are shown for *E. coli* 16S rRNA and human 18S rRNA. Color gradients indicate reactivity levels, with red representing high reactivity, yellow intermediate reactivity, and grey low reactivity.

Alt text: RNA secondary structure visualizations of SSU rRNA with reactivity scores calculated by Modtector and other tools, comparing mutation-based and RT-stop–based structure probing datasets from bacterial and human samples.

Figure S5. Experimental validation of dual signals.
(A) Schematic overview of the experimental design.
(B) Denaturing gel electrophoresis confirming RT-stop signals. Lane 1: DNA marker; lane 2: truncated cDNA detected at the 28S rRNA m1A1322 site; lane 3: full-length cDNA observed in the AlkB-treated control group.
(C) Sanger sequencing chromatogram showing mutation-associated base peaks around the 28S rRNA m1A1322 site.
(D) Quantification of relative base composition at the 28S rRNA m1A1322 position.

Alt text: Experimental validation of dual RNA modification signals, showing the validation workflow, RT-stop detection by denaturing gel electrophoresis, mutation-associated Sanger sequencing peaks, and base composition quantification at the 28S rRNA m1A1322 site.

Table Legends

Table S1. List of tools for benchmark comparison.

Table S2. Runtime and peak memory usage across benchmark datasets.

Independent validation reports

Independent validation report #1

> Assay type: RNA structure / modification probing (E. coli M1 modified sample, DMS-MaPseq-like)
> Species / cell type / sample type: Escherichia coli / M1 mod sample
> Public accession, if available: N/A (local private BAM, ~170 MB)
> Input file type: BAM
> Input file size: 170 MB
> Reference FASTA: 16S_rRNA.fa (first test); M1_EC_regions_add_rRNA.fa (scale-up, 339 contigs)
> Mapped reads: 4,558,199 total mapped; 564,452 to 16S_rRNA; ~307× depth at 16S pos 1
>
> Modtector version: modtector 0.15.4
> Operating system: Darwin 21.5.0 arm64 (macOS Monterey, Apple Silicon)
> CPU model: Apple M1 Max
> Number of CPU cores: 10
> Total memory: 64 GB
> Number of threads used: 4
>
> Command used:
> /usr/bin/time -l modtector count
> -b M1_mod_EC_regions_sorted.bam
> -f 16S_rRNA.fa
> -o modtector_16S_count_v0.15.4.csv
> -s +
> --min-base-qual 20
> -t 4
>
> Elapsed wall-clock time: 0.92 s
> Maximum resident set size: 22,085,632 bytes (~21.1 MB)
> CPU percentage: ~115%
> User time: 1.04 s
> System time: 0.02 s
> Exit status: 0
>
> Output file generated successfully: yes
> Output file size: 79 KB
> First 5 lines of output:
> ChrID,pipe_truncation_Strand,pipe_truncation_ChrPos,rf_mutation_Base,rf_mutation_Count,pipe_truncation_count,depth,effective_depth,rf_mutation_ins,rf_mutation_del,base_A,base_C,base_G,base_T

16S_rRNA,+,1,T,0,307,307,276,0,0,0,0,0,307
16S_rRNA,+,2,T,0,0,307,291,0,0,0,0,0,307
16S_rRNA,+,3,G,0,16,307,295,0,0,0,0,307,0
16S_rRNA,+,4,A,6,26,323,303,0,0,315,1,4,3

Notes / issues / suggestions:

- BAM index (.bai) older than BAM; hts_idx_load3 warning but run succeeded.
- macOS host: used /usr/bin/time -l, sysctl, vm_stat instead of time -v, lscpu, free -h.
- Scale-up to full reference (339 contigs, 10 threads): 4.25 s, ~381 MB RSS, exit 0.

Independent validation report #2

Assay type: RNA structure / modification probing (E. coli M1 modified sample; DMS-MaPseq-like mutation + stop signal assay)

Species / cell type / sample type: Escherichia coli / M1 modified (mod) sample

Public accession, if available: N/A (local private BAM, ~170 MB)

Input file type: BAM

Input file size: 170 MB (M1_mod_EC_regions_sorted.bam)

Reference FASTA: 16S_rRNA.fa (recommended first test, 1.6 KB); M1_EC_regions_add_rRNA.fa (scale-up, 349 KB, 339 contigs)

Approximate sequencing depth / mapped reads: 4,558,199 mapped reads total; 564,452 mapped to 16S_rRNA; ~307× depth at 16S position 1

Modtector version: modtector 0.15.4 (installed via cargo install --git https://github.com/TongZhou2017/modtector)

Operating system: Linux 5.4.0-176-generic x86_64 (Ubuntu; host: shengke)

CPU model: AMD EPYC 7742 64-Core Processor

Number of CPU cores: 256 (255 online)

Total memory: 1.0 TiB (821 GiB available at test time)

Number of threads used: 4 (16S test); 10 (scale-up test)

Command used (recommended first test — 16S rRNA):

```
/usr/bin/time -v modtector count \

  -b M1_mod_EC_regions_sorted.bam \

  -f 16S_rRNA.fa \

  -o modtector_16S_count.csv \

  -s + \

  --min-base-qual 20 \

  -t 4
```

Elapsed wall-clock time: 1.56 s
Maximum resident set size: 22,748 KB (~22.2 MB)
CPU percentage: 123%
User time: 1.87 s
System time: 0.06 s
Exit status: 0

Output file generated successfully: yes
Output file size: 80 KB
First 5 lines of output:

```
ChrID,pipe_truncation_Strand,pipe_truncation_ChrPos,rf_mutation_Base,rf_mutation_Count,pipe_truncation_count,depth,effective_depth,rf_mutation_ins,rf_mutation_del,base_A,base_C,base_G,base_T

16S_rRNA,+,1,T,0,307,307,276,0,0,0,0,0,307

16S_rRNA,+,2,T,0,0,307,291,0,0,0,0,0,307

16S_rRNA,+,3,G,0,16,307,295,0,0,0,0,307,0

16S_rRNA,+,4,A,6,26,323,303,0,0,315,1,4,3
```

Scale-up test (after successful 16S test):

```
/usr/bin/time -v modtector count \

  -b M1_mod_EC_regions_sorted.bam \
```

```
  -f M1_EC_regions_add_rRNA.fa \

  -o modtector_full_ref_count.csv \

  -s + \

  --min-base-qual 20 \

  -t 10
```

Scale-up elapsed wall-clock time: 5.74 s
Scale-up maximum resident set size: 456,744 KB (~446 MB)
Scale-up CPU percentage: 345%
Scale-up exit status: 0
Scale-up output file size: 8.4 MB

Notes / issues / suggestions:

- Independent validation on Linux x86_64 server; data and software installed separately under ~/modtector_validation/.
- Scale-up to 339-contig reference completed successfully with -s + and --min-base-qual 20.
- No errors or crashes observed; output CSV format includes effective_depth column as expected in v0.15.4.

Independent validation report #3

Assay type: RNA structure / modification probing (E. coli M1 modified sample; DMS-MaPseq-like mutation + stop signal assay)

Species / cell type / sample type: Escherichia coli / M1 modified (mod) sample

Public accession, if available: N/A (local private BAM, ~170 MB)

Input file type: BAM

Input file size: 170 MB (M1_mod_EC_regions_sorted.bam)

Reference FASTA: 16S_rRNA.fa (recommended first test, 1.6 KB); M1_EC_regions_add_rRNA.fa (scale-up, 349 KB, 339 contigs)

Approximate sequencing depth / mapped reads: 4,558,199 mapped reads total; 564,452 mapped to 16S_rRNA; ~307× depth at 16S position 1

Modtector version: modtector 0.15.4 (Linux x86_64 binary, same build as independent cargo install on another server)

Operating system: Linux 5.15.0-139-generic x86_64 (Ubuntu 20.04; host: node)

CPU model: Intel(R) Xeon(R) Platinum 8488C

Number of CPU cores: 192

Total memory: 2.0 TiB (1.5 TiB available at test time)

Number of threads used: 4 (16S test); 10 (scale-up test)

Command used (recommended first test — 16S rRNA):

```
/usr/bin/time -v ./modtector count \

  -b M1_mod_EC_regions_sorted.bam \

  -f 16S_rRNA.fa \

  -o modtector_16S_count.csv \

  -s + \

  --min-base-qual 20 \

  -t 4
```

Elapsed wall-clock time: 2.85 s
Maximum resident set size: 22,892 KB (~22.4 MB)
CPU percentage: 110%
User time: 3.08 s
System time: 0.05 s
Exit status: 0

Output file generated successfully: yes
Output file size: 80 KB
First 5 lines of output:

```
ChrID,pipe_truncation_Strand,pipe_truncation_ChrPos,rf_mutation_Base,rf_mutation_Count,pipe_truncation_count,depth,effective_depth,rf_mutation_ins,rf_mutation_del,base_A,base_C,base_G,base_T

16S_rRNA,+,1,T,0,307,307,276,0,0,0,0,0,307
```

16S_rRNA,+,2,T,0,0,307,291,0,0,0,0,0,307

16S_rRNA,+,3,G,0,16,307,295,0,0,0,0,307,0

16S_rRNA,+,4,A,6,26,323,303,0,0,315,1,4,3

Scale-up test (after successful 16S test):

```
/usr/bin/time -v ./modtector count \

  -b M1_mod_EC_regions_sorted.bam \

  -f M1_EC_regions_add_rRNA.fa \

  -o modtector_full_ref_count.csv \

  -s + \

  --min-base-qual 20 \

  -t 10
```

Scale-up elapsed wall-clock time: 11.81 s
Scale-up maximum resident set size: 364,988 KB (~356 MB)
Scale-up CPU percentage: 210%
Scale-up exit status: 0
Scale-up output file size: 8.4 MB
Scale-up first 5 lines of output:

ChrID,pipe_truncation_Strand,pipe_truncation_ChrPos,rf_mutation_Base,rf_mutation_Count,pipe_truncation_count,depth,effective_depth,rf_mutation_ins,rf_mutation_del,base_A,base_C,base_G,base_T

BOGIMF_01972_Eup0_Edown0,+,1,T,0,1,1,1,0,0,0,0,0,1

BOGIMF_01972_Eup0_Edown0,+,2,A,0,0,1,1,0,0,1,0,0,0

BOGIMF_01972_Eup0_Edown0,+,3,A,0,0,1,1,0,0,1,0,0,0

BOGIMF_01972_Eup0_Edown0,+,4,T,0,0,1,0,0,0,0,0,0,1

Notes / issues / suggestions:

- Independent validation under user account on a separate server; data uploaded independently to ~/modtector_validation/.

- Source compile failed on this host (missing fontconfig dev libs; user not in sudoers); validation completed with modtector 0.15.4 Linux x86_64 binary.
- Scale-up to 339-contig reference succeeded; output size 8.4 MB consistent with other runs.

Independent validation report #4

Assay type: RNA structure probing (SHAPE-MaP paired-end test dataset)
Species / cell type / sample type: Custom IVT RNA / NAI-treated sample
Public accession, if available: N/A (local private FASTQ, subsampled for testing)
Input file type: BAM
Input file size: 9.5 MB
Reference FASTA: 7.fasta
Mapped reads: N/A; test BAM generated from subsampled paired-end FASTQ using Bowtie2 + samtools

Modtector version: modtector 0.15.4
Operating system: Ubuntu 24.04 LTS under WSL2
CPU model: Intel(R) Core(TM) i5-14400F
Number of CPU cores: 16
Total memory: 15 GiB
Number of threads used: 8

Command used:
/usr/bin/time -v modtector count
-b test.sorted.bam
-f 7.fasta
-o result/test_count.csv
-t 8

Elapsed wall-clock time: 0.64 s
Maximum resident set size: 20,708 KB (~20.2 MB)
CPU percentage: 120%
User time: 0.73 s
System time: 0.04 s
Exit status: 0

Output file generated successfully: yes
Output file size: 640 KB

First 5 lines of output:
ChrID,pipe_truncation_Strand,pipe_truncation_ChrPos,rf_mutation_Base,rf_mutation_Count,pipe_truncation_count,depth,rf_mutation_ins,rf_mutation_del,base_A,base_C,base_G,base_T
7,+,1,A,0,2,1,0,0,1,0,0,0

> 7,-,1,A,7,0,9,0,0,2,6,0,1
> 7,+,2,T,1,0,2,0,0,1,0,0,1
> 7,-,2,T,8,9,24,0,0,3,5,0,16
>
> Notes / issues / suggestions:
>
> - Successfully compiled and tested on Ubuntu 24.04 LTS under WSL2.
> - Input BAM was generated from paired-end FASTQ using Bowtie2 and samtools.
> - A subsampled FASTQ dataset was used for functional validation.
> - Native Windows installation encountered dependency issues; switching to WSL2 provided a working environment.
> - modtector count completed successfully and generated the expected CSV output.

871
