# Supplementary material for: Modtector: ultra-fast modification signal mining on mapped sequencing reads
Source: Bioinformatics. 2026 Aug 20;42(9):btag627. doi: 10.1093/bioinformatics/btag627 (PMC13533567; doi:10.1093/bioinformatics/btag627)
Supplement: Supplementary file 1 [file btag627_supplementary_data.zip › Supplementary Figures.pdf]

Figure S1

A

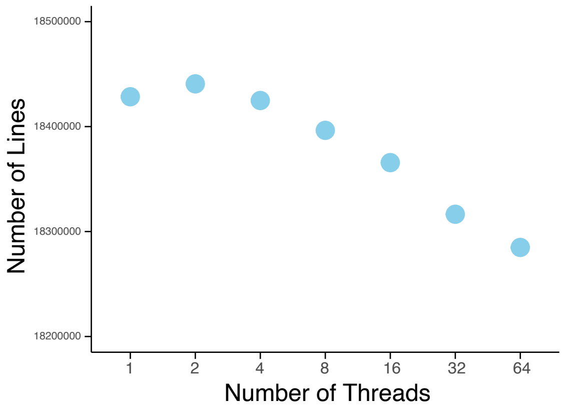
18500000
18400000
18300000
18200000
Number of Lines
1
2
4
8
16
32
64
Number of Threads

Figure S2

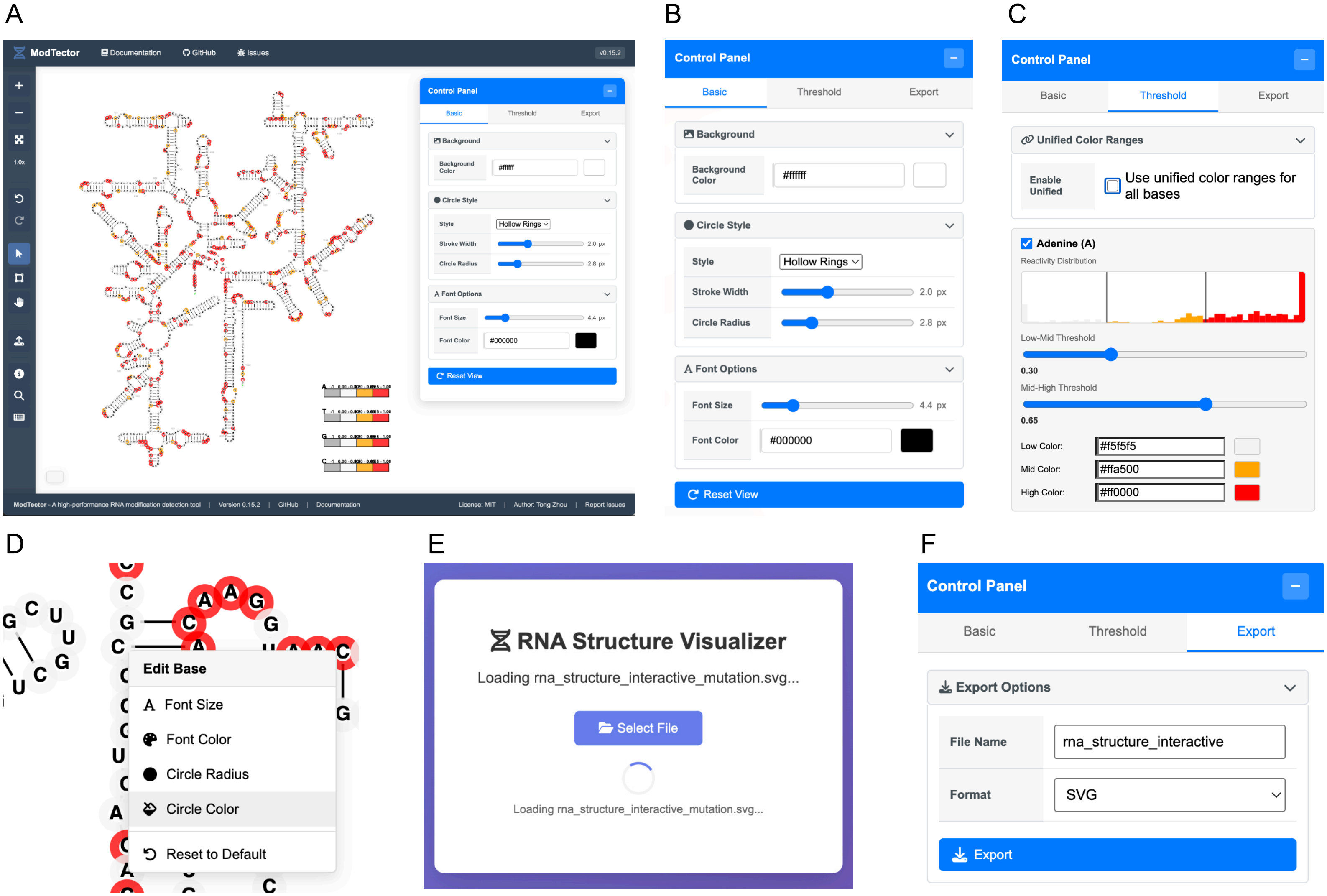
A
B
C
D
E
F
ModTector
Documentation
GitHub
Issues
v0.15.2
ModTector - A high-performance RNA modification detection tool | Version 0.15.2 | GitHub | Documentation
License: MIT | Author: Tong Zhou | Report Issues
Control Panel
Basic
Threshold
Export
Background
Background Color
#ffffff
Circle Style
Style
Hollow Rings
Stroke Width
2.0 px
Circle Radius
2.8 px
Font Options
Font Size
4.4 px
Font Color
#000000
Reset View
Unified Color Ranges
Enable Unified
Use unified color ranges for all bases
Adenine (A)
Reactivity Distribution
Low-Mid Threshold
0.30
Mid-High Threshold
0.65
Low Color:
#f5f5f5
Mid Color:
#ffa500
High Color:
#ff0000
Edit Base
Font Size
Font Color
Circle Radius
Circle Color
Reset to Default
RNA Structure Visualizer
Loading rna_structure_interactive_mutation.svg...
Select File
Loading rna_structure_interactive_mutation.svg...
Export Options
File Name
rna_structure_interactive
Format
SVG
Export

Figure S3

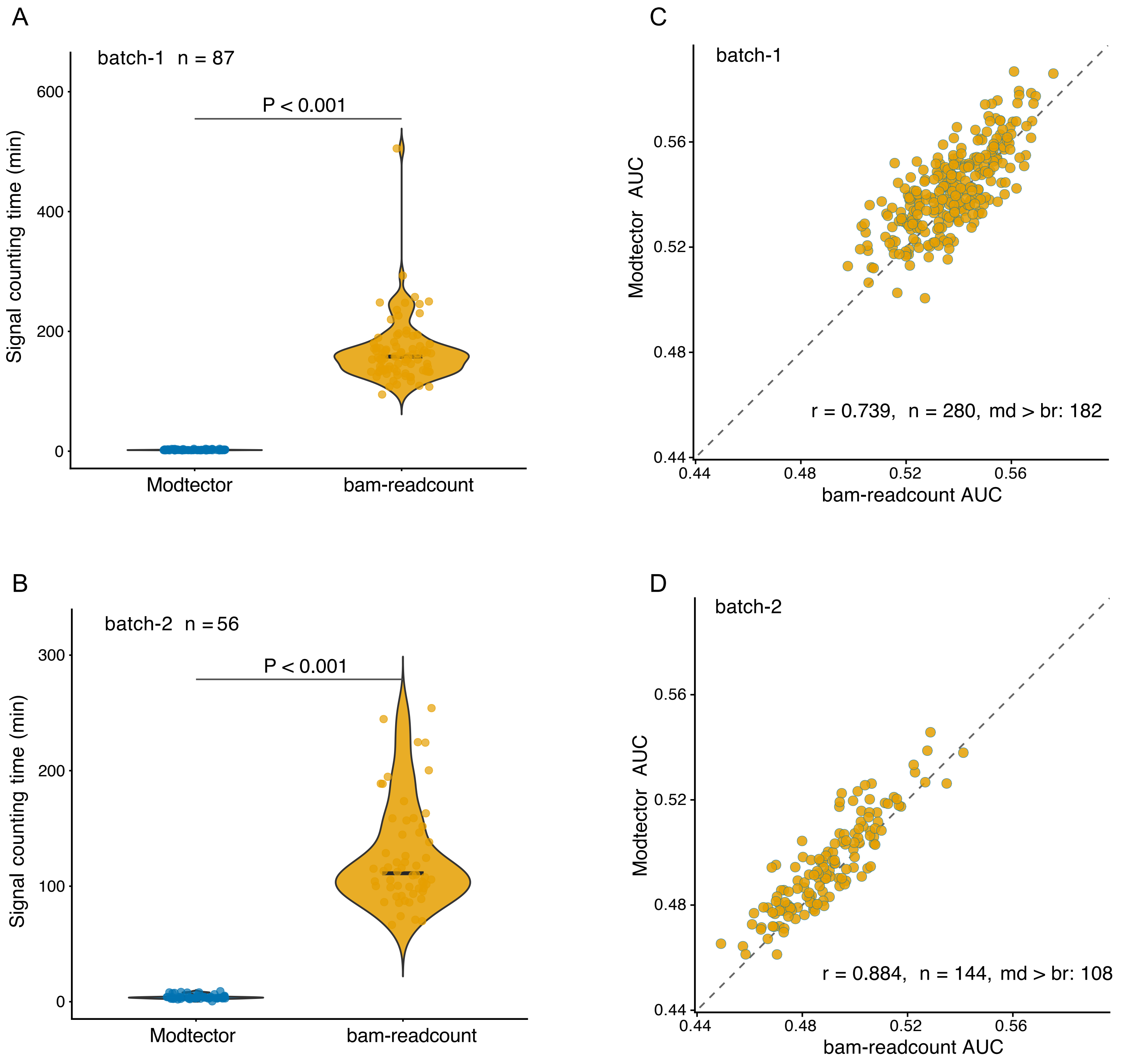
A
batch-1 n = 87
P < 0.001
Signal counting time (min)
600
400
200
0
Modtector
bam-readcount
B
batch-2 n = 56
P < 0.001
Signal counting time (min)
300
200
100
0
Modtector
bam-readcount
C
batch-1
Modtector AUC
0.56
0.52
0.48
0.44
r = 0.739, n = 280, md > br: 182
0.44
0.48
0.52
0.56
bam-readcount AUC
D
batch-2
Modtector AUC
0.56
0.52
0.48
0.44
r = 0.884, n = 144, md > br: 108
0.44
0.48
0.52
0.56
bam-readcount AUC

Figure S4

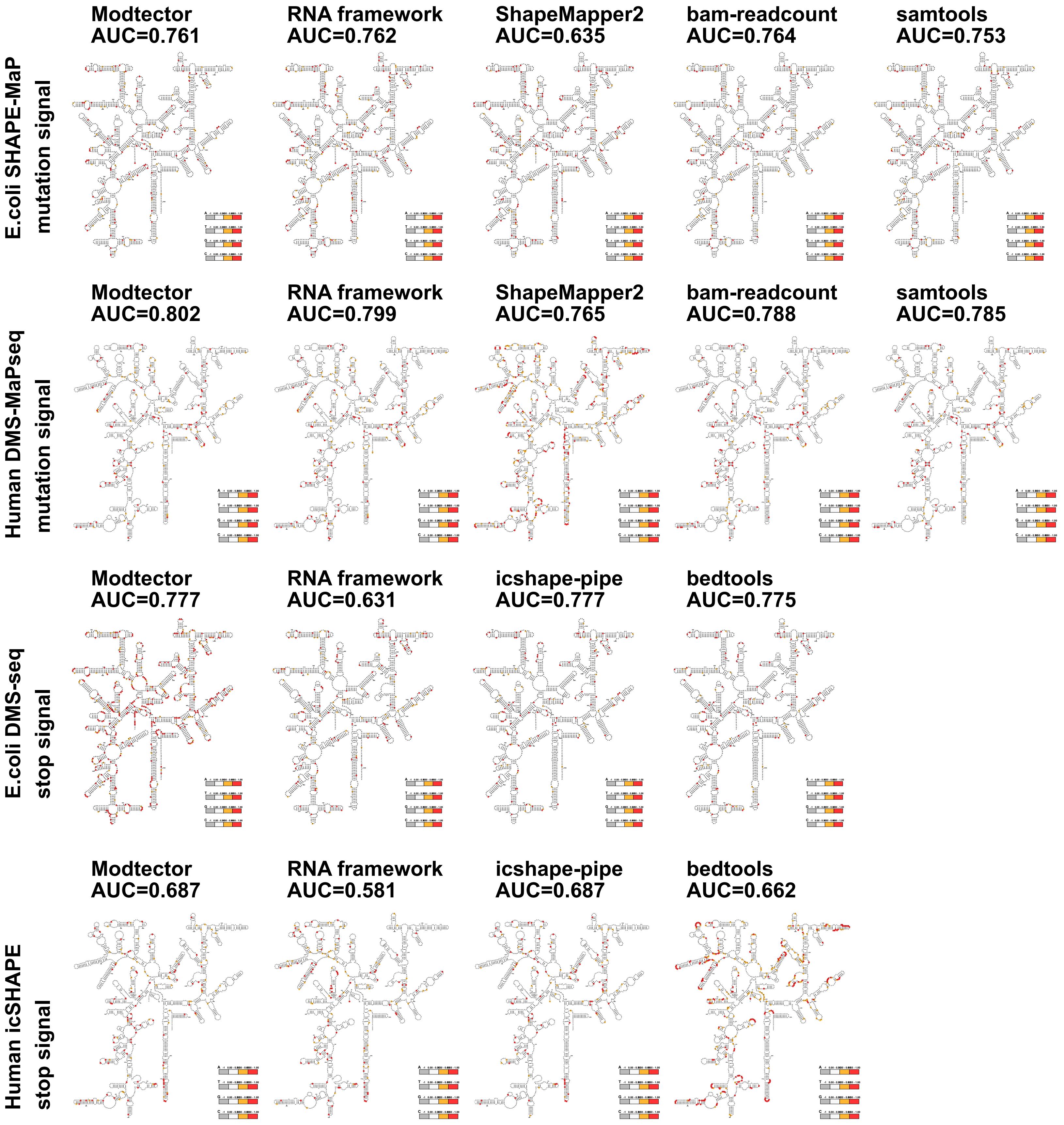
E.coli SHAPE-MaP
mutation signal
Modtector
AUC=0.761
RNA framework
AUC=0.762
ShapeMapper2
AUC=0.635
bam-readcount
AUC=0.764
samtools
AUC=0.753
Human DMS-MaPseq
mutation signal
Modtector
AUC=0.802
RNA framework
AUC=0.799
ShapeMapper2
AUC=0.765
bam-readcount
AUC=0.788
samtools
AUC=0.785
E.coli DMS-seq
stop signal
Modtector
AUC=0.777
RNA framework
AUC=0.631
icshape-pipe
AUC=0.777
bedtools
AUC=0.775
Human icSHAPE
stop signal
Modtector
AUC=0.687
RNA framework
AUC=0.581
icshape-pipe
AUC=0.687
bedtools
AUC=0.662

Figure S5

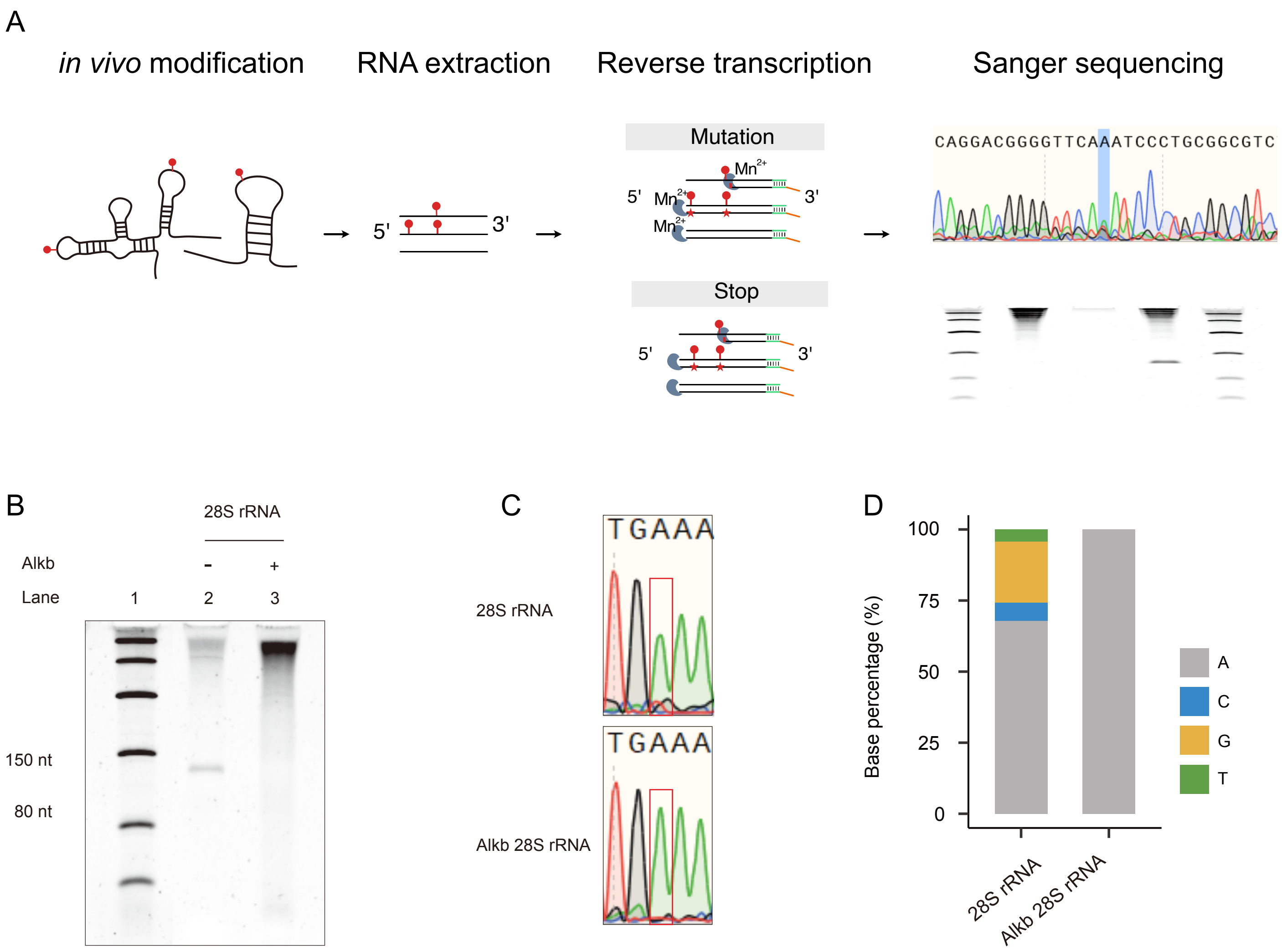

A
in vivo modification
RNA extraction
Reverse transcription
Sanger sequencing
5'
3'
Mutation
Mn2+
5'
3'
Stop
5'
3'
CAGGACGGGGTTCAAATCCCTGCGGCGTC
B
28S rRNA
Alkb
-
+
Lane
1
2
3
150 nt
80 nt
C
28S rRNA
TGAAA
Alkb 28S rRNA
TGAAA
D
Base percentage (%)
100
75
50
25
0
28S rRNA
Alkb 28S rRNA
A
C
G
T
